# Causal Associations between Serum Urea and Cancer: A Mendelian Randomization Study

**DOI:** 10.3390/genes12040498

**Published:** 2021-03-29

**Authors:** Yandi Sun, Jingjia Li, Zihao Qu, Ze Yang, Xueyao Jia, Yindan Lin, Qian He, Lihong Zhang, Yan Luo

**Affiliations:** 1 Department of Biochemistry and Cancer Institute of the Second Affiliated Hospital, Zhejiang University School of Medicine, Hangzhou 310058, China; 11418070@zju.edu.cn (Y.S.); lijingjia@zju.edu.cn (J.L.); zyang2015@zju.edu.cn (Z.Y.); 21818609@zju.edu.cn (X.J.); 13610059151@163.com (Y.L.); 11518073@zju.edu.cn (Q.H.); 642052480@zju.edu.cn (L.Z.); 2 Key Laboratory of Cancer Prevention and Intervention of China National Ministry of Education, Hangzhou 310058, China; 3 Department of Orthopedic Surgery, The Second Affiliated Hospital, Zhejiang University School of Medicine, Hangzhou 310058, China; qu_z.h@zju.edu.cn; 4 Orthopedic Research Institute of Zhejiang University, Hangzhou 310058, China

**Keywords:** Mendelian randomization, serum urea, renal cell carcinoma, breast cancer, prostate cancer

## Abstract

Urea is largely derived from the urea cycle reactions through hepatic detoxification of free ammonia and cleared by urination, and the serum urea level is a crucial medical indicator for measuring the kidney function in patients with nephropathy; however, investigative revelations pointing to the serum urea level as a risk factor for cancer are very scarce, and relevant studies are restricted by potential biases. We aimed to explore the causal relationships of the serum urea level with cancer development by focusing on renal cell carcinoma (RCC) using the Mendelian randomization (MR) analyses. Summary estimates were collected from the inverse-variance weighted (IVW) method based on six single nucleotide polymorphisms (SNPs). The selected SNPs related to the serum urea were obtained from a large genome-wide association study (GWAS) of 13,312 European participants. The summary statistics of RCC were also available from public databases (IARC, *n* = 5219 cases, *n* = 8011 controls). Sensitivity analyses included the weighted median and MR-Egger methods. Serum urea was inversely associated with RCC in females (effect = 1.93; 95% CI: 1.24 to 3.01; *p* = 0.004) but exhibited null association with RCC in males, breast cancer (BRCA) in both genders and prostate cancer (PCa) in males. Similar conclusions were also drawn from the weighted median and MR-Egger. These findings reveal an intriguing link between serum urea and cancer risks for the very first time. Without ambiguity, the serum urea is causatively related to RCC specifically in females, although the mechanism(s) by which urea is involved in RCC development remains to be experimentally/clinically investigated. Our studies may well provide novel insights for RCC diagnosis, intervention and/or therapy.

## 1. Introduction

Over the past several decades, the incidence of kidney cancers appears to be increasing worldwide, which constitutes the deadliest malignant tumor of the urinary system. Early diagnosis remains the key: the five-year survival rate for patients in stage I is about 93%, whereas for patients in stage II/III is 72.5%; for patients in stage IV, however, the five-year survival rate drastically drops to 12% [1]. Circa 80–90% of kidney cancers eventually evolved into renal cell carcinoma (RCC). It is well known that RCC is metastatic, heterogeneous, somewhat hereditary, and often resistant to chemotherapy and radiation therapy. RCC is categorized into clear renal cell carcinoma (70–80%), papillary renal cell carcinoma (10–15%), chromophobe renal cell carcinoma (5%), hereditary cancer syndromes (5%), collecting duct carcinoma (1%), Chromosome Xp11.2 translocation (1%) and other types, which at an end-stage are typically associated with three clinical symptoms: cruenturesis, osphyalgia and renal mass building [2,3,4]. If these symptoms all occur, the renal cell carcinoma is characterized to be at an end-stage that already misses the best therapy period [5]. In addition, the overall incidence rate of RCC in males is higher than in females because the androgen receptor (AR) promotes the progression of RCC in males [6,7]. Steroid hormone receptor-related tumors also include breast cancer (BRCA) and prostate cancer (PCa), of which the BRCA is one of the most malignant tumors in the female population. It is established that most BRCA have classical biomarkers such as estrogen receptor, progesterone receptor, and human epidermal growth receptor-2 [8]. But the androgen receptor (AR) is also found to be expressed in the majority of breast tumor tissues, and AR is known to participate in the pathology and development of BRCA [9,10]**.** With respect to PCa, it is a common disease in elderly males and can also be regulated by the AR-related signaling pathway [11,12]. Therefore, to some extent, RCC, BRCA, and PCa are the cancer types that are all related to AR.

Epidemiological studies unraveled relative risks for renal cell carcinoma, including genetic and environmental factors; e.g., a growing body of evidence exhibits high susceptibility of patients, with inherited conditions such as the Von Hippel Lindau (VHL) and Birt–Hogg–Dubé (BHD) syndromes, to renal cell carcinoma [13,14]. Genetic risk factors aside [15,16]**,** other risk factors such as smoking, obesity, hypertension, and end-stage renal diseases are also definitely (causatively) related to renal cell carcinoma [17,18]**.** In fact, end-stage renal diseases are found to play dominant roles in the neoplastic development of renal cell carcinoma; patients, especially those undergoing hemodialysis, are strongly advised to avoid a high-protein diet in order to reduce the urea production that constitutes a nitrogen burden. This is consistent with the fact that urea is an indispensable biomarker in clinical diagnosis and is strongly associated with kidney function [19,20]**.** Glomerulonephritis, nephritis, and renal failure result in the accumulation of urea in the blood, and excessive urea is harmful to the human liver, kidney, and lung. Moreover, the elevated urea induces systemic inflammation and promotes cardiovascular disease. But there have been no clinical trials to investigate the association between urea and RCC or other cancers, and despite the above-indicated findings, the causal relationship between urea and cancers (renal cell carcinoma, BRCA and PCa) remains elusive and needs to be explored through new/novel approaches.

Mendelian Randomization (MR) analyses have become a popular method to determine the influence of exposures on diverse diseases [21,22,23]. It is difficult to infer the causal relationship between exposures such as urea and clinical outcomes in many cases, and randomized controlled trials cannot be performed due to ethics issues. However, MR analyses are designed to solve the above problems and directly verify the causal relationship. More importantly, MR analyses take advantage of the single nucleotide polymorphism (SNP), which in conjunction with external elements can avoid confounding factors and other potential complications [24,25].

To date, no study relies on MR analyses to demonstrate the role of urea in renal cell carcinoma and other cancers such as breast cancer. In this report, we used the MR approach to explore the causal effects of serum urea levels on renal cell carcinoma (RCC), breast cancer (BRCA), and prostate cancer (PCa), and the potential associations among them were further determined. The results offer novel insights that may pave the way for better RCC diagnosis, intervention and therapy.

## 2. Materials and Methods

### 2.1. Study Design and Data Sources

**Figure 1:**
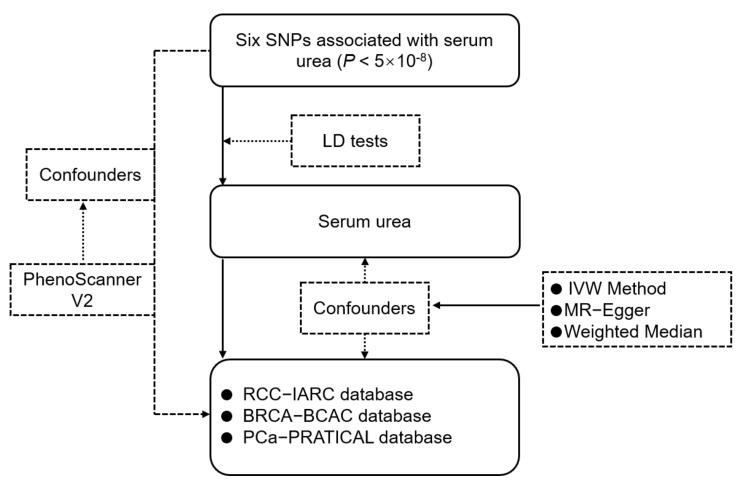
The flowchart of Mendelian Randomization analysis of serum urea and risk of three types of cancer. The study is based upon the assumption that the SNPs are related to serum urea, but not associated with the risk of three types of cancer on serum urea and confounders.

We designed a two-sample Mendelian Randomization approach in this study (Figure 1). It is based on the assumption that instrumental variables are related to serum urea, but independent of the risk of cancer and cofounders.

The instrumental variables (IVs) were obtained from the Lifelines Cohort Study of 13,312 participants, of European ancestry, on serum urea [26]**.** The participants were genotyped using the SNP array and called by GenomeStudio (San Diego, CA, USA). SNPs were removed if the call rate was less than 95%, the *p*-value was above 10^−6^ and the minor allele frequency was less than 1%. Information of data on the association of SNPs with serum urea and the association of SNPs with renal cell carcinoma, breast cancer and prostate cancer were obtained from the GWAS database [27,28,29]**.**

### 2.2. Genetic Association with Outcomes

Summary-level data for the association of each IV with renal cell carcinoma were extracted from the sex-specific GWAS meta-analysis for men (3227 cases and 4916 controls) and women (1992 cases and 3095 controls) of European ancestry, in which the top findings were replicated in two additional cohorts of European origin of men and women (2261 cases and 5852 controls, 1399 cases and 1575 controls, respectively) [27] (Table 1).

The breast cancer genome-wide association study consisted of 89,677 European individuals (46,785 cases and 42,892 non-cases) collected from 41 studies using a custom genotyping array, but it was restricted to females [28] (Table 1). 

Summary genetic data for prostate cancer were obtained from GWAS’s publicly meta-analyses [29] (Table 1). The prostate cancer association study included 140,254 men of European ancestry (79,148 cases and 61,106 non-cases).

### 2.3. Selection of Instrumental Variables

We employed the published instrumental variables associated with serum urea from GWAS [26]. The six selected SNPs were within or near the THBS3, LPP, ADAMTS9-AS2, PTGER4, RSPO3 and POU2AF1 genes and reached a significant threshold (*p*-value < 5 × 10^−8^) for their association with serum urea. The selected genetic instruments were located in five genomic regions. Detailed information is shown in Table 2.

### 2.4. Statistical Methods

In this study, we employed three different statistical methods, namely the inverse-variance weighted (IVW) method**,** Mendelian Randomization–Egger (MR-Egger) and the weighted median method.

For genetics variables validation, aiming to verify the proposition that IVs affect cancers only through serum urea, we employed the PhenoScanner V2 database to ensure that the IVs were not directly associated with cancers [30,31]. To ensure that the six IVs are independently (r^2^ < 0.02) associated with serum urea, we searched the linkage disequilibrium (LD)-link website (https://ldlink.nci.nih.gov, 15 September 2020) to conduct linkage disequilibrium tests. Finally, six SNPs were selected as the IVs to predict the serum level of urea.

In our study, the IVW method provides the estimate of a causal effect of serum urea on three types of cancer, which is based on the assumption that the selected SNPs are validated. Given that ð_XY_ represents the causal effect of serum urea (X) on the three types of cancer (Y), ð_XY_ can be estimated through the equation as ð_XY_ = ð_ZY_/ð_ZX_, where ð_ZY_ represents the effects of SNPs (Z) on cancer (Y) and ð_ZX_ represents the effects of SNPs (Z) on the serum urea. We tested the SNPs validation and performed the IVW method with random-effect model to explain potential heterogeneity.

The weighted median method (WME) draws a consistent estimate from at least 50% information from valid genetic SNPs [32,33] for sensitivity analyses. In addition, the MR–Egger regression method is applied to provide valid estimates for causal association [34,35,36], which is usually used in the random-effects model. Confidence intervals [36] and *p*-values can be obtained from t-distribution. It should be noted that the validity of MR-Egger regression estimates required SNPs strength independent of the pleiotropic effects on the outcome.

Statistical analyses were conducted by R (version 3.6.1) and R package (Mendelian Randomization) [31], and the two-sided *p*-values (<0.05) were considered as the threshold of significance.

## 3. Results

Six SNPs were published in the GWAS database to be predictive of serum urea level: rs914615 (THBS3), rs4686914 (LPP), rs998394 (ADAMTS9-AS2), rs11954639 (PTGER4), rs2503107 (RSPO3), and rs2003313 (POU2AF1) (Table 2). Two SNPs (rs4686914 and rs998394) were located in the same chromosome; we performed a linkage disequilibrium assay to estimate the instrumental variables (Supplementary Table S1). None of the SNPs selected was excluded from the MR analyses, and no key confounders and pleiotropic effects associated with RCC, BRCA, and PCa were identified. The associations between serum urea and RCC, BRCA, and PCa were summarized in Supplementary Tables S2–S4.

### 3.1. Association of Urea Variants with RCC, BRCA, and PC

**Figure 2:**
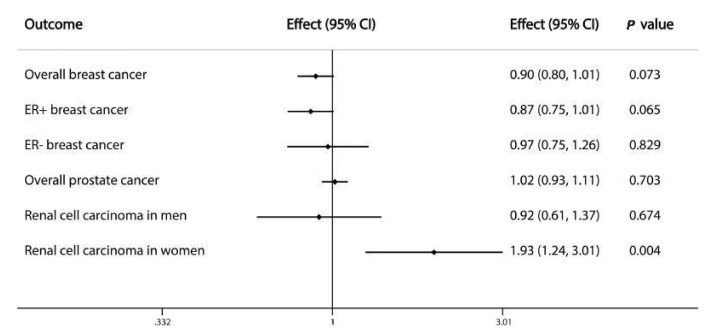
Forest plot of causal associations of serum urea on overall BRCA, overall PCa and RCC in female and male. Effect: The causal association; 95% CI: 95% confidence interval; *p*-value: the significance of the instrumental variants.

The IVW identified the associations of six serum urea-related SNPs with RCC, BRCA and PCa. We found that genetically instrumented serum urea was not associated with overall BRCA (effect: 0.898; 95% CI: 0.797–1.010; *p*-value: 0.073) including ER+ (effect: 0.869; 95% CI: 0.748–1.010; *p*-value: 0.065) and ER- (effect: 0.971; 95% CI: 0.747–1.263; *p*-value: 0.829) subtypes (Figure 2 and Supplementary Table S5) or PCa (effect: 1.016; 95% CI: 0.935–1.105; *p*-value: 0.703) (Figure 2 and Supplementary Table S5). However, of the six serum urea-related SNPs, the SNP (rs4686914) located in chromosome 3q exhibited most strongly associated with female RCC. There was a significant relationship between serum urea levels and female RCC (effect: 1.930; 95% CI: 1.238–3.009; *p*-value: 0.004) rather than male RCC (effect: 0.917; 95% CI: 0.613–1.372; *p*-value: 0.674) (Figure 2 and Supplementary Table S5). These results indicated that elevated serum urea levels were dangerous for renal cell carcinoma in females.

### 3.2. Sensitivity Analyses

To assess the genetically valid SNPs’ pleiotropic effects, we used different approaches with different assumptions, such as MR-Egger and the weighted median. MR Egger’s test suggested that the instrument variants are independent of the outcomes if the intercept reached zero baselines. In our study, MR-Egger regression exhibited strong evidence that directional pleiotropic effects were unlikely to bias the results of overall BRCA (intercept: −0.023; *p* = 0.094), RCC in females (intercept: 0.047; *p* = 0.382), RCC in males (intercept: −0.043; *p* = 0.456) and PCa (intercept: 0.004; *p* = 0.732) (Supplementary Table S6). Moreover, the weighted median showed directionally similar estimates in overall BRCA (β: −0.029; *p* = 0.683), RCC in females (β: 0.644; *p* = 0.029), RCC in males (β: −0.275; *p* = 0.238) and PCa (β: 0.035; *p* = 0.523) (Supplementary Table S6).

**Figure 3:**
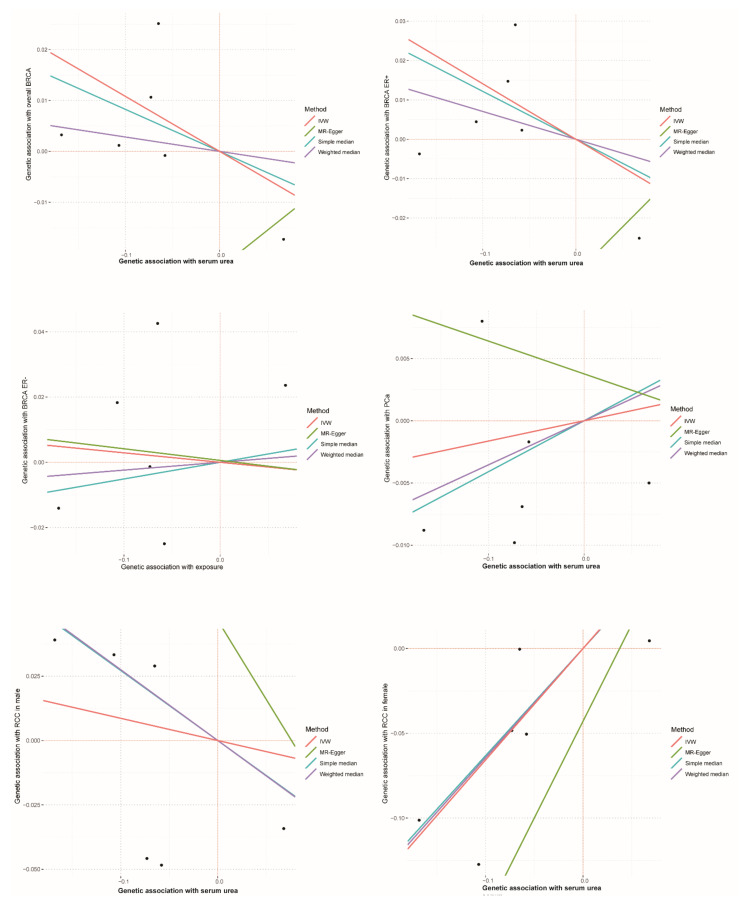
Scatter plots of genetics associated with cancer plotted against genetics associated with serum urea. The estimates for six SNPs on overall BRCA, BRCA ER+, BRCA ER−, PCa, RCC in male and RCC in females were shown.

Figure 3 shows scatter plots of genetics association with three types of cancer against genetics association with serum urea, which directly revealed the causal effects for each SNP on overall BRCA, BRCA ER+, BRCA ER-, PCa, RCC in males and RCC in females. Detailed information is shown in Supplementary Table S7. In addition, the physiological traits of the six SNPs did not directly relate to BRCA, RCC, and PCa (Supplementary Table S8). These sensitivity analyses above supported the notion that serum urea is associated with RCC in females but not with RCC in males, BRCA, and PCa.

## 4. Discussion

In this study, the causal effects between serum urea and cancer risks such as RCC, BRCA, and PCa were examined through MR methodology. Our findings support the notion that there was no valid evidence to support the association between serum urea and the risks of BRCA (ER+/ER−) or PCa. However, serum urea was strongly related to renal cell carcinoma, and the higher concentration of serum urea exerted negative effects in females rather than in males, which implicates the higher risk of RCC. Moreover, the entire set of results are robust to plenty of sensitivity analyses.

Serum urea is the end product of protein metabolism, filtered through the glomerulus, and finally excreted from the human body. Studies to evaluate the influence of serum urea on the carcinogenic occurrence have been extremely scarce and inconclusive [37]. For instance, based on a case of 80 workers, EI Far et al. [38] measured levels of three cancer biomarkers (carcinoembryonic antigen, α-fetoproteins, and prostate-specific antigen) in male workers exposed to urea for eight years, but all the urea values maintained at a range of physiological condition. Through a case-control study of 1841 workers in a nitrogen products (including urea) industry, Marsh et al. [39] assessed nitrogen products’ associations with bladder cancer risk. However, there was no elaboration about urea alone; thus, the effects of urea still remain elusive. Overall, no study has specifically demonstrated a potential role of urea in cancer risks. Due to diverse cancer types, small sample sizes, and different analysis methods, no consistent conclusion has been drawn. We used the MR approach to reveal that higher levels of genetically predicted serum urea were directly proportional to the incidence of RCC in females, and no association of serum urea with BRCA or PCa was identified. Our study strongly points to a causal relationship between serum urea and RCC prognosis.

Potential mechanisms underlying the higher risk of serum-urea-related RCC in females rather than in males may include three categories. First, there are obvious physiological differences between females and males in humans. A female kidney volume is smaller, and the number of the nephron is less compared with males. More importantly, the nephron is the functional unit to produce urine containing urea, and this means that females excrete the serum urea slower than males under the same condition. Besides, the glomerular filtration rates in males were faster than in females (92.0 vs. 88.1 mL/min/1.73 m^2^, *p* < 0.0001), which leads to the accumulation of urea [40,41]. Second, sex steroids such as estrogen and androgen are other factors to be tested. It is reported that androgen plays a protective role in the male kidney [42,43]. Thus, congenital differences may dictate degrees of urea accumulation in blood, which in females may more easily reach above a threshold with a high incidence of RCC. Third, animal studies have demonstrated that excessive urea gave rise to DNA damage and chromosome 3p fragmentation [44,45], which causes the incidence of RCC. However, rs4686914 is located in chromosome 3q and is not influenced by chromosome 3p. In view of this, we predict that the rs4686914 locus may play an important role in the pathology of RCC in females via urea. However, these propositions need to be verified by cell assays and animal experiments in the future. 

There are several strengths and limitations in this study. In terms of strengths, to our knowledge, this is the first Mendelian Randomization study that evaluated the causal role of serum urea in three cancers. The sample size is relatively large based on the GWAS databases. The six SNPs were not related to other traits, suggesting that the positive association was not affected by the confounders between urea-related SNPs and cancers. In terms of limitations, we may need to explore more SNPs associated with blood urea to assess the risks of cancers. Moreover, GWAS’s database about different types of cancer is minimal. Hence, our study cannot screen all the cancers. In addition, despite the novelty of our MR analyses, experimental/clinical verification for a link between serum urea and RCC neoplastic development is not yet available; more intriguingly, why females have higher risks of RCC in relation to serum urea level, as our analyses unambiguously suggest, remains mysterious. Furthermore, it is worth mention that the population we studied was limited to European individuals; the conclusion might not be directly applicable to other populations such as those in Asia. Despite these limitations, however, uncovering biological mechanisms linking serum urea levels to sex-specific cancer development, animal models included, should be a worthy goal.

## 5. Conclusions

In summary, this MR study shows that the levels of serum urea are positively associated with risks of RCC specifically in females and, more importantly, sex differences in the relationship of serum urea and risks of RCC were determined. Little evidence supports the causal effects of serum urea on BRCA and PCa. This study offers novel insights into the role(s) of serum urea in the development of RCC in females. The serum urea level is a novel indicator/target for assessing renal cell carcinoma.

## Figures and Tables

**Table 1:** The characteristics of GWAS studies on the outcomes.

Outcome.	SNPs	Consortium	Total Population	Cases/Controls	Ethnicity	References
Total BRCA	6	BCAC	89,677	46,785/42,892	European	Genome-wide association analysis of more than 120,000 individuals identifies 15 new susceptibility loci for breast cancer. PubMed id: 25751625
Overall PCa	6	PRATICAL	140,254	79,148/61,106	European	Association analyses of more than 140,000 men identify 63 new prostate cancer susceptibility loci. PubMed id: 29892016
RCC in female	6	IARC	5087	1992/3095	European	Sex-specific associations in genome-wide association analysis of renal cell carcinoma PubMed id: 31231134
RCC in male	6	IARC	8143	3227/4916	European	Sex-specific associations in genome-wide association analysis of renal cell carcinoma. PubMed id: 31231134

**Table 2:** Characteristics of SNPs for serum urea level.

SNP	Chromosome	Type	Nearby Gene	Serum Urea			
				EA	β	SE	*p*-Value
rs914615	1	Intronic	THBS3	A	0.068	0.012	4 × 10^−9^
rs4686914	3	Intergenic	LPP	T	–0.107	0.013	3 × 10^−17^
rs998394	3	ncRNA/intronic	ADAMTS9-AS2	A	–0.058	0.011	4 × 10^−7^
rs11954639	5	Intergenic	PTGER4	T	–0.168	0.027	6 × 10^−10^
rs2503107	6	Intronic	RSPO3	C	–0.065	0.013	5 × 10^−7^
rs2003313	11	intergenic	POU2AF1	T	–0.073	0.012	1 × 10^−9^

EA: effect allele; β: the effect on the serum urea; SE: standard error.

## Data Availability

This work used publicly available data, and the data sources are described in Materials and Methods. Further details are available from the corresponding authors on a reasonable request.

## Supplementary Materials

The following are available online at https://www.mdpi.com/article/10.3390/genes12040498/s1, Table S1: The linkage disequilibrium analyses of selected SNPs. Table S2: The association information of serum urea SNPs with overall BRCA and subtypes in European individuals. Table S3: The association information of serum urea SNPs with overall PCa in European individuals. Table S4: The association information of serum urea SNPs with RCC in females and males. Table S5: The raw data of forest plot about serum urea’s causal associations on all the outcomes. Table S6: Weighted median and MR-Egger analysis for genetic associations between serum urea level and all the outcomes. Table S7: The detailed information of scatter plots. Table S8: Related traits of serum urea SNPs.

## Abbreviations

MR	Mendelian Randomization
BRCA	Breast Cancer
PCa	Prostate Cancer
RCC	Renal Cell Carcinoma
SNP	Single Nucleotide Polymorphism
GWAS	Genome-Wide Association Studies
IVW	Inverse-Variance Weighted
IV	Instrumental Variable
