# Supplementary material for: Causal Associations between Serum Urea and Cancer: A Mendelian Randomization Study
Source: Genes (Basel). 2021 Mar 29;12(4):498. doi: 10.3390/genes12040498 (PMC8066321; doi:10.3390/genes12040498)
Supplement: Supplementary file 1 [file genes-12-00498-s001.zip › genes-1130322(2).docx]

Supplementary Materials

Table 1. The linkage disequilibrium analyses of selected SNPs

| RS number | rs4686914 | rs998394 |
| --- | --- | --- |
| rs4686914 | 1.0 | 0.014 |
| rs998394 | 0.014 | 1.0 |

The two SNPs are in the same chromosome 3. r^2^ < 0.05.

| SNP | EA | Overall BRCA | | BRCA (ER^+^) | | BRCA (ER^-^) | |
| --- | --- | --- | --- | --- | --- | --- | --- |
|  |  | Βeta (SE) | P value | Βeta (SE) | P value | Βeta (SE) | P value |
| rs914615 | A | -0.017 (0.001) | 0.072 | -0.025 (0.012) | 0.029 | 0.024 (0.019) | 0.21 |
| rs4686914 | T | 0.001 (0.010) | 0.91 | 0.004 (0.012) | 0.72 | 0.018 (0.020) | 0.37 |
| rs998394 | A | -0.001 (0.012) | 0.95 | 0.002 (0.014) | 0.87 | -0.025 (0.023) | 0.28 |
| rs11954639 | T | 0.003 (0.018) | 0.86 | -0.004 (0.0213) | 0.86 | -0.014 (0.035) | 0.69 |
| rs2503107 | C | 0.025 (0.001) | 0.011 | 0.029 (0.012) | 0.014 | 0.043 (0.019) | 0.03 |
| rs2003313 | T | 0.011 (0.011) | 0.31 | 0.015 (0.013) | 0.24 | -0.001 (0.021) | 0.95 |

Table 2. The association information of serum urea SNPs with overall BRCA and subtypes in European individuals.

EA = effect allele; β = per allele effect on SD units; SE = standard error; P value = p-value for the genetic association.

Table 3. The association information of serum urea SNPs with overall PCa in European individuals.

| SNP | EA | Overall PCa | |
| --- | --- | --- | --- |
|  |  | Βeta (SE) | P value |
| rs914615 | A | -0.005 (0.008) | 0.072 |
| rs4686914 | T | 0.008 (0.009) | 0.91 |
| rs998394 | A | -0.002 (0.008) | 0.95 |
| rs11954639 | T | -0.009 (0.015) | 0.86 |
| rs2503107 | C | -0.007 (0.008) | 0.011 |
| rs2003313 | T | -0.010 (0.008) | 0.31 |

EA = effect allele; β = per allele effect on SD units; SE = standard error; P value = p-value for the genetic association.

Table 4. The association information of serum urea SNPs with RCC in female and male.

| SNP | EA | Association with RCC  in Female | | Association with RCC  in Male | |
| --- | --- | --- | --- | --- | --- |
|  |  | Beta (SE) | P value | Beta (SE) | P value |
| rs914615 | A | 0.005 (0.043) | 0.916 | -0.034 (0.035) | 0.328 |
| rs4686914 | T | -0.127 (0.046) | 0.006 | 0.033 (0.037) | 0.370 |
| rs998394 | A | -0.051 (0.043) | 0.240 | -0.048 (0.034) | 0.157 |
| rs11954639 | T | -0.101 (0.078) | 0.192 | 0.039 (0.062) | 0.530 |
| rs2503107 | C | -0.000 (0.043) | 0.992 | 0.029 (0.034) | 0.397 |
| rs2003313 | T | -0.048 (0.043) | 0.262 | -0.046 (0.035) | 0.187 |

EA = effect allele; β = per allele effect on SD units; SE = standard error; P value = p-value for the genetic association.

Table 5. The raw data of forest plot about casual associations of serum urea on all the outcomes.

|  | Overall BRCA | BRCA  (ER^+^) | BRCA  (ER^-^) | Overall  PCa | RCC in  Female | RCC in  Male |
| --- | --- | --- | --- | --- | --- | --- |
| Estimate  (SE) | -0.108  (0.060) | -0.141  (0.076) | -0.029  (0.134) | 0.016  (0.043) | 0.658  (0.226) | -0.086  (0.205) |
| 95% CI | [-0.226,0.010] | [-0.290,0.008] | [-0.292,0.234] | [-0.067,0.100] | [0.214,1.102] | [-0.489,0.316] |
| P Value | 0.073 | 0.065 | 0.829 | 0.703 | 0.004 | 0.674 |

SE = standard error; CI = confidence interval; P value = p-value for the casual association.

Table 6. Weighted median and MR-Egger analysis for genetic associations between serum urea level and all the outcomes.

| Method | Weighted median | MR-Egger | |
| --- | --- | --- | --- |
|  |  | Estimate | Intercept |
| Overall BRCA |  |  |  |
| Estimate (95% CI) | -0.029  [-0.164, 0.107] | 0.147  [-0.167, 0.463] | -0.023  [-0.050, 0.004] |
| P value | 0.683 | 0.362 | 0.094 |
| BRCA (ER^+^) |  |  |  |
| Estimate (95% CI) | -0.071  [-0.234, 0.093] | 0.238  [-0.138, 0.615] | -0.034  [-0.067, -0.002] |
| P value | 0.397 | 0.214 | 0.036 |
| BRCA (ER^-^) |  |  |  |
| Estimate (95% CI) | 0.024  [-0.246, 0.294] | -0.036  [-0.934, 0.863] | 0.001  [-0.076, 0.077] |
| P value | 0.862 | 0.938 | 0.988 |
| Overall PCa |  |  |  |
| Estimate (95% CI) | 0.035  [-0.073, 0.144] | -0.026  [-0.284, 0.231] | 0.004  [-0.018, 0.025] |
| P value | 0.523 | 0.841 | 0.732 |
| RCC in female |  |  |  |
| Estimate (95% CI) | 0.644  [0.066, 1.222] | 1.142  [-0.207, 2.492] | 0.047  [-0.058, 0.151] |
| P value | 0.029 | 0.097 | 0.382 |
| RCC in male |  |  |  |
| Estimate (95% CI) | -0.275  [-0.732, 0.182] | -0.614  [-1.866, 0.638] | -0.043  [-0.156, 0.070] |
| P value | 0.238 | 0.337 | 0.456 |

CI = confidence interval; P value = p-value of the causal estimate.

Table 7. The detailed information of scatter plots

| Overall BRCA | | | | |
| --- | --- | --- | --- | --- |
| Method | Estimate | Std Error | 95% CI | P-value |
| Simple median | -0.082 | 0.074 | [-0.227, 0.062] | 0.262 |
| Weighted median | -0.028 | 0.069 | [-0.164, 0.107] | 0.683 |
| IVW | -0.108 | 0.060 | [-0.226, 0.010] | 0.073 |
| MR-Egger  (intercept) | 0.147 | 0.161 | [-0.169, 0.463] | 0.362 |
|  | -0.023 | 0.014 | [-0.050, 0.004] | 0.094 |

| BRCA ER+ | | | | |
| --- | --- | --- | --- | --- |
| Method | Estimate | Std Error | 95% CI | P-value |
| Simple median | -0.122 | 0.091 | [-0.301, 0.058] | 0.183 |
| Weighted median | -0.071 | 0.083 | [-0.234, 0.093] | 0.397 |
| IVW | -0.141 | 0.076 | [-0.290, 0.008] | 0.065 |
| MR-Egger  (intercept) | 0.238 | 0.192 | [-0.138, 0.615] | 0.214 |
|  | -0.034 | -0.066 | [-0.066, -0.002] | 0.036 |

| BRCA ER- | | | | |
| --- | --- | --- | --- | --- |
| Method | Estimate | Std Error | 95% CI | P-value |
| Simple median | 0.051 | 0.137 | [-0.218, 0.320] | 0.710 |
| Weighted median | 0.024 | 0.138 | [-0.246, 0.294] | 0.862 |
| IVW | -0.029 | 0.134 | [-0.292, 0.234] | 0.829 |
| MR-Egger  (intercept) | -0.036 | 0.458 | [-0.934, 0.863] | 0.938 |
|  | 0.001 | 0.039 | [-0.076, 0.077] | 0.988 |

| BRCA PCa | | | | |
| --- | --- | --- | --- | --- |
| Method | Estimate | Std Error | 95% CI | P-value |
| Simple median | 0.041 | 0.057 | [-0.070, 0.152] | 0.471 |
| Weighted median | 0.035 | 0.055 | [-0.073, 0.144] | 0.523 |
| IVW | 0.016 | 0.043 | [-0.067, 0.100] | 0.703 |
| MR-Egger  (intercept) | -0.026 | 0.131 | [-0.284, 0.231] | 0.841 |
|  | 0.004 | 0.011 | [-0.018, 0.025] | 0.732 |

| RCC in male | | | | |
| --- | --- | --- | --- | --- |
| Method | Estimate | Std Error | 95% CI | P-value |
| Simple median | -0.272 | 0.232 | [-0.727, 0.183] | 0.242 |
| Weighted median | -0.275 | -0.275 | [-0.732, 0.182] | 0.238 |
| IVW | -0.086 | 0.205 | [-0.489, 0.316] | 0.674 |
| MR-Egger  (intercept) | -0.614 | 0.639 | [-1.866, 0.638] | 0.337 |
|  | 0.047 | 0.053 | [-0.058, 0.151] | 0.382 |

| RCC in female | | | | |
| --- | --- | --- | --- | --- |
| Method | Estimate | Std Error | 95% CI | P-value |
| Simple median | 0.633 | 0.298 | [0.048, 1.217] | 0.034 |
| Weighted median | 0.644 | 0.295 | [0.066, 1.222] | 0.029 |
| IVW | 0.034 | 0.226 | [0.214, 1.102] | 0.004 |
| MR-Egger  (intercept) | 1.142 | 0.689 | [-0.207, 2.492] | 0.097 |
|  | -0.043 | 0.058 | [-0.156, 0.070] | 0.456 |

CI = confidence interval; P value = p-value of the causal estimate.

Table 8. Related traits of serum urea SNPs.

| SNP | Chromosome: Position | Trait | Beta | P value |
| --- | --- | --- | --- | --- |
| rs914615 | 1 | Hematocrit | -0.024 | 1.18E-11 |
| rs914615 | 1 | Hemoglobin concentration | -0.021 | 5.21E-09 |
| rs914615 | 1 | log Urinary albumin creatinine ratio | -0.029 | 6.30E-06 |
| rs914615 | 1 | Serum urate | 0.033 | 1.13E-07 |
| rs914615 | 1 | Crohns disease | NA | 2.20E-06 |
| rs914615 | 1 | Inflammatory bowel disease | -0.081 | 2.77E-06 |
| rs914615 | 1 | Urinary albumin to creatinine ratio | 0.03 | 7.00E-06 |
| rs914615 | 1 | Impedance of leg left | -0.029 | 5.21E-09 |
| rs914615 | 1 | Impedance of leg right | -0.012 | 1.77E-07 |
| rs914615 | 1 | Leg fat-free mass left | -0.012 | 7.72E-07 |
| rs914615 | 1 | Leg fat-free mass right | 0.007 | 5.23E-06 |
| rs914615 | 1 | Leg predicted mass left | 0.007 | 5.73E-06 |
| rs914615 | 1 | Leg predicted mass right | 0.007 | 5.96E-06 |
| rs914615 | 1 | Mouth or teeth dental problems: dentures | 0.007 | 5.66E-06 |
| rs914615 | 1 | Impedance of leg right | -0.005 | 4.87E-08 |
| rs4686914 | 3 | Blood urea nitrogen BUN | NA | 2.87E-21 |
| rs4686914 | 3 | Metabolite levels | -0.024 | 3.00E-21 |
| rs4686914 | 3 | Blood urea nitrogen | NA | 3.00E-21 |
| rs11954639 | 5 | Bipolar disorder and schizophrenia | NA | 8.44E-06 |
| rs11954639 | 5 | Crohns disease | NA | 5.90E-08 |
| rs2003313 | 11 | Hemoglobin concentration | -0.016 | 9.13E-06 |
| rs2503107 | 6 | Hematocrit | 0.022 | 5.04E-10 |
| rs2503107 | 6 | Hemoglobin concentration | 0.023 | 5.08E-11 |
| rs2503107 | 6 | High light scatter reticulocyte count | 0.017 | 2.08E-06 |
| rs2503107 | 6 | Red blood cell count | 0.023 | 1.88E-10 |
| rs2503107 | 6 | Reticulocyte count | 0.018 | 3.04E-07 |
| rs2503107 | 6 | Hip circumference adjusted for BMI | -0.018 | 8.50E-08 |
| rs2503107 | 6 | Waist circumference | 0.018 | 7.10E-08 |
| rs2503107 | 6 | Waist hip ratio in physically active females | 0.045 | 4.60E-16 |
| rs2503107 | 6 | Waist hip ratio | 0.037 | 2.60E-13 |
| rs2503107 | 6 | High density lipoprotein | -0.019 | 1.69E-09 |
| rs2503107 | 6 | Triglycerides | 0.017 | 1.83E-06 |
| rs2503107 | 6 | Fracture of forearm | -0.001 | 2.54E-09 |
| rs2503107 | 6 | Heel bone mineral density | 0.065 | 1.00E-96 |
| rs2503107 | 6 | Hip circumference | -0.014 | 5.21E-09 |
| rs2503107 | 6 | Varicose veins of lower extremities | -0.001 | 2.41E-07 |

The traits were found to be related with urea-associated SNPs. The results were available from PhenoScanner V2 website (http://www.phenoscanner.medschl.cam.ac.uk/).
